# Refining Scraps—Gold, Platinum, Etc.

**Published:** 1865-01

**Authors:** W. P. Haywood


REFINING SCRAPS—GOLD, PLATINUM, ETC.—*By W. P. Haywood*.—We will suppose all the silver is removed from the scraps and fillings by the process described in the last number of the *Quarterly*. We have now the gold and platina in combination to separate, with still a small quantity of iron, which for reasons already given, will not be acted upon by nitric acid. The metals left after removing the silver, should be washed free from nitric acid, then treated to a dose of muriatic acid for three or four hours, aided by a gentle heat, over a sand bath. This will remove all the iron. Then, after washing until free from acid, dissolve the metals left, in four parts muriatic, and one part nitric acid.

To regain the gold, which is the most important, as the small amount of platina in dentists' scraps will not pay for the trouble of separating and collecting it, make a saturated solution of protosulphate of iron, (green copperas,) filter it, and add to the gold solution until there ceases to fall down a dark muddy brown precipitate. In this process the gold and platina solution must be previously evaporated until nearly dry; then add distilled water, and then by degrees the solution of iron. By evaporating the solution of gold we get rid of an excess of nitric acid, which would otherwise convert the protosulphate of iron into persulphate by the action of nitric acid on the former, giving off nitrous oxide gas. The persulphate of iron will not precipitate gold to any extent. If, after evaporation, the gold solution is too weak to form a vigorous precipitate, add a small quantity of muriatic acid.

A solution of any metal should be as strong in that acid which facilitates its being precipitated by its proper precipitant as it will possibly bear, to give the best results. A weak solution gives a light floculent minute precipitate. A strong solution, if not overheated, gives a coarse one. It is only necessary to wash the brown precipitate from acid, and after partly drying, melt with borax.—*Dental Quarterly.*

